# A simple integrated primary health care based model for detection of diabetic retinopathy in resource-limited settings in Pakistani population

**DOI:** 10.12669/pjms.325.10955

**Published:** 2016

**Authors:** Ali Jawa, Muhammad Zaman Khan Assir, Syed Hunain Riaz, Rashin Chaudhary, Farooq Awan, Javed Akram

**Affiliations:** 1 Prof. Ali Jawa, MD, MPH, DABIM, FACE. Shaheed Zulfiqar Ali Bhutto Medical University, PIMS, Islamabad, Pakistan; 2 Muhammad Zaman Khan Assir, FCPS. Shaheed Zulfiqar Ali Bhutto Medical University, PIMS, Islamabad, Pakistan; 3 Syed Hunain Riaz, FCPS. Wilshire Cardiovascular and Endocrine Center of Excellence (WILCARE), Lahore, Pakistan; 4 Rashin Chaudhary, MPH. Fred Hollows Foundation, Islamabad, Pakistan; 5 Farooq Awan, MPH, M.COM, MBA. Fred Hollows Foundation, Islamabad, Pakistan; 6 Prof. Javed Akram, MD, MRCP, FRCP, FRCP, FACP, FACC, FASIM. Shaheed Zulfiqar Ali Bhutto Medical University, PIMS, Islamabad, Pakistan

**Keywords:** Diabetes Mellitus, Diabetic Retinopathy, Pakistan, Blindness, Lady Health Workers

## Abstract

**Objective:**

To find out prevalence of Diabetic Retinopathy in general population of three districts in Pakistan.

**Methods:**

A community based cross-sectional survey was conducted in three large districts of Pakistan namely Rawalpindi in Punjab, Peshawar in Khyber Pakhtoonkhwa and Hyderabad in Sindh between January 2013 and August 2015. Lady Health Workers identified individuals at high risk for diabetes based on pre-defined criteria. High risk population was tested for dysglycemia. Fundoscopic evaluation for evidence of DR was performed in all individuals with a random blood glucose >190mg/dl. Individuals with the evidence of DR were referred to affiliated tertiary care ophthalmology departments.

**Results:**

A total of 42,629 individuals reported at the project sites and 63% (n=26,859) were female. Fifty one percent (n=21,989) individuals met high risk criteria. Out of these 21,989 individuals, dysglycemia was found in 3,869 (17.6%). Fundoscopy showed evidence of DR in 1,042 (27%) individuals. Amongst high risk population, dysglycemia was significantly more common in females as compared to males. The frequency of DR in dysglycemic patients was comparable across both gender groups.

**Conclusion:**

The prevalence of DR in Pakistani population is alarmingly high. This preventable cause of blindness is largely undiagnosed in our population and a simple integrated model based on primary health care facilities can help identify and treat a large population of DR patients.

## INTRODUCTION

Non communicable diseases (NCDs) kill 38 million people each year and Diabetes Mellitus (DM) is the fourth leading cause of NCD deaths. According to WHO, the estimated global prevalence of DM among adults above 18 years of age is about 9%.^1^ In 2012, an estimated 1.5 million deaths were directly caused by diabetes and more than 80% of diabetes deaths occurred in low- and middle-income countries^2^. Pakistan in a lower middle income country and with a population of 182 million Pakistan is the 6^th^ most populous country of the world.

According to the National Diabetes Survey, more than 10% of adult population of Pakistan has diabetes.^3^-^5^ Diabetic retinopathy is the leading cause of new cases of blindness in adults aged 20-74 years.^6^ The prevalence of DR is reported to be up to 40% in a pooled analysis of population based eye surveys in the US.^7^ Diabetes and diabetic retinopathy are under-diagnosed in Pakistan. National Diabetes Survey showed that for each known case of DM, there were approximately two cases of undiagnosed DM and three cases of impaired glucose tolerance in the population.^8^ Moreover, evaluation for retinopathy in diabetic patients is not routinely done. In a clinical based study conducted on 223 adult Pakistani diabetic patients with >3 years history of diabetes, more than 70% had never been assessed for diabetic retinopathy (DR).^9^ Despite high prevalence of DM, true population-based data on prevalence of DR is lacking. The prevalence of DR in Pakistani diabetic population is reported to be 15.7% in a small population-based study (n=108).^10^

In order to study the prevalence of DR and decrease the incidence of preventable blindness in three districts of Pakistan, we adopted an integrated cascade model of health education, community participation, screening, confirmation of diagnosis and treatment. This model integrated primary health care facilities with specialized treatment centers. Here we report the results of a large population-based study on prevalence of DR.

## METHODS

This cross-sectional and observational study with non-probability purposive sampling was conducted as a part of Fred Hollows Foundation’s project on Health System Strengthening for DM and DR to prevent blindness. The Fred Hollows Foundation is a non-government organization which seeks to eradicate avoidable blindness in developing countries and to improve the health of Indigenous Australians. Fred Hollows Foundation has been operational in Pakistan since 2008 and has funded and supported several projects including DR project.

## Ethical approval

All procedures performed in studies involving human participants were in accordance with the ethical standards of the institutional and/or national research committee and with the 1964 Helsinki declaration and its later amendments or comparable ethical standards. Informed consent was obtained from all individual participants included in the study.

The DR study was conducted in three districts of Pakistan namely Rawalpindi in Punjab, Peshawar in Khyber Pakhtoonkhwa and Hyderabad in Sindh between January 2013 and August 2015. The purpose of the project was to test the Health Systems Strengthening (HSS) approach to identify, treat / manage and refer patients to appropriate level and also building the capacity of community and primary level health facilities staff to undertake this assignment and continue it beyond project life. The project objectives were to identify DM in high risk population and check DR in patients found with impaired glucose levels, raise awareness regarding DM & DR among the targeted population, and build the capacity and create a link between community, primary and tertiary level health facilities for the provision of quality of care both for DM and DR. This project was implemented by The Fred Hollows Foundation with the support of government tertiary level hospitals at two locations namely Hayatabad Medical Complex (HMC) in Peshawar, Liaquat University of Medical & Health Sciences (LUMHS) Hyderabad and an NGO, Layton Rahmatullah Benevolent Trust (LRBT) Rawalpindi. The study was approved by Ethical Review Committee of Shaheed Zulfiqar Ali Bhutto Medical University, Islamabad and is in accordance with declaration of Helsinki.

Each district has a number of basic health units (BHUs) (primary level health facility in Pakistan) and about 15-25 Lady Health Workers (LHWs) are affiliated with each BHU. A lady health supervisor (LHS) supervises LHWs at each BHU. There are 19 BHUs in district Hyderabad, 50 BHUs in district Peshawar and 98 BHUs in district Rawalpindi.

The project team at each of three project site was comprised of a Social Organizer (Team leader) and qualified Optometrist. The Social Organizer was oriented on project management whereas Optometrist was specifically trained to test blood glucose level, undertake detailed eye examination and differentiate between normal and abnormal retina in a dilated eye using direct ophthalmoscope. A brief course curriculum was developed for Lady Health Workers (LHWs) training. A cascade training methodology was adopted to train LHWs in all three districts. In the first phase a basic training on diabetes, its complications and high risk factors for diabetes was delivered to District Master Trainer and Lady Health Supervisor (LHSs) by a qualified ophthalmologist with the assistance of project team. The District Master Trainer and LHSs then delivered training to LHWs in their respective communities. LHWs training were done in batches after dividing district into sub-district and then sub-district in a cluster of 8 to 10 BHUs in each cluster. LHWs visited door to door to educate the families and identify individuals with high risk features for DM. Individuals were defined high risk for diabetes if they had two or more of the following high risk features: over 40 years of age, family history for diabetes, frequent urination, obesity, slow wound healing and unintentional weight loss.

Individuals meeting above-mentioned high-risk criteria were pricked by LHWs for capillary blood glucose level using glucometer at BHUs. A glucometer of same specifications was used across all screening sites throughout the project and was regularly calibrated. Individuals with random blood glucose of ≥ 190 mg/dL were referred to the BHUs for measurement of blood glucose by laboratory testing. Once diabetes was confirmed at BHU by the Medical Officer after obtaining venous blood samples, individuals were referred for detailed ophthalmoscopic examination as well as to BHU physician for diabetes management.

Patient history was recorded by the Optometrist on the Basic Health Unit (BHU) were further evaluated for diabetic retinopathy using ophthalmoscope. All individuals with the evidence of DR were referred to Ophthalmology department of affiliated tertiary care facilities. The frequencies and percentages were calculated for categorical variables. Gender differences in the prevalence of dysglycemia and DR were calculated by applying Chi Square statistics. A p value of < 0.05 was considered statistically significant.

## RESULTS

A total of 42,629 individuals reported at the project sites during year 2013-15 and 63% (n=26,859) were female. Most individuals were from Punjab (56.8%) followed by Sindh (36.9%) and KPK (6.3%) [Table-I]. Out of all individuals reporting at the project sites (BHUs), majority (56.2%) reported on their own (n=23,957) while 34.4% (n=14,664) were referred by LHWs, 5.5% (n=2,334) were referred by general practitioners (GPs) or family physicians and 7.4% (n=3,159) were referred by Lady Health Supervisors (LHS) from Basic Health Units (BHUs). A significantly higher proportion of males self-reported to project sites as compared to females (58.7% [9,264 out of 15770] vs. 54.7% [14,693 out of 26,859] respectively; p value < 0.001). On the other hand, a higher proportion of females was referred by LHWs (35.8% [9,627 out of 26,859] vs. 31.9% [5,036 out of 15,770] respectively; p value <0.001), LHSs (8.7% [2,341 out of 26,859] vs. 5.2% [818 out of 15,770] respectively; p value <0.001) and GPs (5.8% [1,553 out of 26,859] vs. 4.9% [781 out of 15,770] respectively; p value <0.001).

**Table-I:** District-wise cascade analysis of Diabetic Retinopathy data.

	*Rawalpindi (Punjab)*	*Hyderabad (Sindh)*	*Peshawar (KPK)*
*Individuals who visited BHUs*			
Total	24,214	15,718	2,697
Male	9,456	5,638	676
Female	14,758	10,080	2,021
*Met high risk criteria*			
Total	7,999	11,631	2,359
Male	3,063	4,874	569
Female	4,936	6,757	1,790
*Random blood glucose >190 mg/dl*			
Total	1,666	1,347	854
Male	516	452	201
Female	1,150	895	653
*Evidence of diabetic retinopathy*			
Total	519	223	300
Male	161	65	76
Female	358	158	224
*Reported at tertiary care centers*			
Total	143	143	257
Male	50	43	76
Female	93	100	181

**Fig.1:**
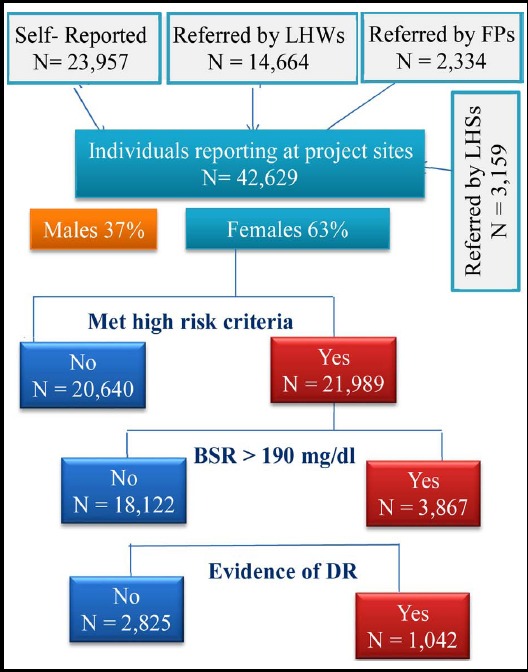
Algorithm for screening individuals for DR using the Primary Health Care model.

Out of 42,629 individuals visiting the project sites, 21,989 (51.2%) met the high-risk criteria and were tested for capillary random blood glucose using glucometer [Fig.1]. A random blood glucose concentration of >190 mg/dl was found in 3,869 (17.59%) individuals. A significantly higher percentage of males met high risk criteria as compared to females (53.9% [8,506 out of 15,770] vs. 50.2% [13,483 out of 26,859] respectively; p value <0.001). On the other hand, significantly higher percentage of females fulfilling high risk criteria were found to have blood glucose >190 mg/dl (20% [2,698 out of 13,483] vs. 13.7% [1,164 out of 8,506] respectively; p value <0.001). Fundoscopy performed on all 3,869 individuals with blood glucose >190 mg/dl showed evidence of diabetic retinopathy in 1,042 (26.9%) individuals. The frequency of diabetic retinopathy in individuals with dysglycemia was comparable across both genders (27.4% [740 out of 2,698 females] vs. 25.8% [302 out of 1,169 males]; p value = 0.32). A difference in prevalence of DR was observed across three districts being highest in KPK (35.1%) followed by Punjab (31.1%) and Sindh (16.5%) [Table-I].

All 1,042 individuals with the fundoscopic evidence of diabetic retinopathy (DR) were referred to tertiary care level ophthalmology centers for the management of diabetic retinopathy; however, only 543 individuals (52.1%) reported to these centers. A higher percentage of males with evidence of DR reported to tertiary care centers as compared to females (56.9% [172 out of 302] vs. 50.1% [371 out of 740] respectively); however, the difference was just statistically significant (p=0.05). All patients were thoroughly examined by consultant ophthalmologists. Fundoscopy and slit lamp examination were performed in all 543 patients and all were found to have DR. The data on grading of DR was not retrievable. Additionally, Fundus Fluorescein Angiography (FFA) was performed in 20 patients.

A number of treatment modalities were offered to patients with the evidence of diabetic retinopathy. Out of 543 DR patients, laser photocoagulation was performed in 147 (27.1%) patients. Intravitreal injection of bevacizumab was given in 498 (91.7%) patients. Many patients received both laser and injections that is why the total count exceeding number of patients referred.

## DISCUSSION

Analysis of pooled data from a number of population-based studies of 22,896 individuals from 35 studies in the U.S., Australia, Europe, and Asia showed an overall prevalence of 35% for any DR. It is estimated that there are approximately 93 million people with DR, 17 million with proliferative DR, 21 million with diabetic macular edema, and 28 million with VTDR worldwide. Longer diabetes duration and poorer glycemic and blood pressure control are strongly associated with DR.^11^ Ethnicity appears to be an important risk factor for DR. The prevalence of DR is reported to be highest amongst African Americans (49.6%) and lowest amongst Asians (19.9%).^11^

Our population-based study shows that overall prevalence of DR is high (27%) in Pakistani population as compared to other Asian populations. Prior studies on prevalence of DR in Pakistani population have shown conflicting results. A small community-based study from Northern Karachi (Sindh) showed a 15.7% prevalence of DR.^10^ However the sample size was small and these results cannot be generalized to other sub-populations. For example, our study finds different prevalence of DR across provinces, being highest in KPK (35%) and lowest in Sindh (16.5%). This difference in prevalence of DR across distinct sub-populations may be explained on the basis of genetic differences. However larger well designed studies are warranted to study this effect. A number of clinic-based studies have shown a prevalence of DR of 26%^12^ and 57%^9^ in Karachi (Sindh) and 21% in Lahore (Punjab)^13^ respectively. From our and previously published population-based studies, it is evident that clinic-based studies overestimate the prevalence of DR and these results cannot be generalized. Although the prevalence of dysglycemia was higher amongst females as compared to males who self-reported high risk features, the prevalence of DR was comparable across both the genders. This finding is consistent with prior studies that show no significant effect of gender on prevalence of DR.

Our study provides a simple and practical model for identification of diabetic retinopathy and prevention of a significant number of blindness cases by integrating primary level health care services with tertiary care facilities. LHWs and LHSs can serve as key population educators and mobilizers and can contribute significantly towards overall community health.

The design of this study was kept simple in order to make at practical in resource-limited settings. Although ADA defines diabetes as a random venous blood glucose of ≥200 mg/dl, a cutoff of 190 mg/dl for random capillary blood glucose was used in our study to enhance the sensitivity of our screening process. The glucometers used had a confidence range of ±10 mg/dl and thus a cutoff of 190 mg/dl was used to identify most DR cases. Glucometer was used by LHWs due to its ease of application. Optometrists were trained to identify any DR for uniformity. The type of DR was not documented at BHUs. Since all these cases were further evaluated by consultant ophthalmologists, the stage of DR was characterized at the specialized centers; however, unfortunately, this individual-level data was not available.

## CONCLUSION

A concerted approach towards early identification and management of DR is required in order to decrease the burden of preventable blindness. A system of training of LHWs and other paramedical staff at BHUs, screening of individuals for DM and DR at primary care center and referral to tertiary care facilities for management of DR can best serve the purpose. With the ever increasing burden of DM and DR, there is an urgent need for setting up even more tertiary level ophthalmology centers in Pakistan.
